# Transdiagnostic Cognitive Behavioral Therapy Versus Treatment as Usual in Adult Patients With Emotional Disorders in the Primary Care Setting (PsicAP Study): Protocol for a Randomized Controlled Trial

**DOI:** 10.2196/resprot.6351

**Published:** 2016-12-23

**Authors:** Antonio Cano-Vindel, Roger Muñoz-Navarro, Cristina Mae Wood, Joaquín T Limonero, Leonardo Adrián Medrano, Paloma Ruiz-Rodríguez, Irene Gracia-Gracia, Esperanza Dongil-Collado, Iciar Iruarrizaga, Fernando Chacón, Francisco Santolaya

**Affiliations:** ^1^ PsicAP Research Group Complutense University of Madrid Madrid Spain; ^2^ Faculty of Psychology Complutense University of Madrid Madrid Spain; ^3^ Faculty of Psychology University of Valencia Valencia Spain; ^4^ Faculty of Psychology Autonomous University of Barcelona Barcelona Spain; ^5^ Faculty of Psychology Universidad Siglo 21 Córdoba Argentina; ^6^ Fuenlabrada Primary Care Center Health Service of Madrid Madrid Spain; ^7^ Faculty of Psychology Catholic University of Valencia Valencia Spain; ^8^ Faculty of Social Work Complutense University of Madrid Madrid Spain; ^9^ Spanish Association of Psychologists Madrid Spain; ^10^ Malva-Rosa Mental Health Service Valencia Spain

**Keywords:** anxiety, depression, somatization, treatment as usual, cognitive behavioral therapy, quality of life, primary care, emotional disorders, transdiagnostic therapy, medically unexplained symptoms, common mental health disorders

## Abstract

**Background:**

Demand for primary care (PC) services in Spain exceeds available resources. Part of this strong demand is due to the high prevalence of emotional disorders (EDs)—anxiety, depression, and somatic symptom disorders—and related comorbidities such as pain or chronic illnesses. EDs are often under- or misdiagnosed by general practitioners (GPs) and, consequently, treatment is frequently inadequate.

**Objective:**

We aim to compare the short- and long-term effectiveness of group-delivered transdiagnostic cognitive behavioral therapy (TD-CBT) versus treatment as usual (TAU) in the treatment of EDs in the PC setting in Spain. We also aim to compare the effect of these treatments on disability, quality of life, cognitive-emotional factors, and treatment satisfaction.

**Methods:**

Here we present the study design of a two-arm, single-blind, randomized controlled trial (N=1126) to compare TAU to TD-CBT for EDs. TAU will consist primarily of pharmacological treatment and practical advice from the GP while TD-CBT will be administered in seven 90-minute group sessions held over a period ranging from 12 to 14 weeks. Psychological assessments are carried out at baseline (ie, pretreatment); posttreatment; and at 3-, 6-, and 12-month follow-up. The study is conducted in approximately 26 PC centers from the National Health System in Spain.

**Results:**

This study was initiated in December 2013 and will remain open to new participants until recruitment and follow-up has been completed. We expect all posttreatment evaluations to be completed by December 2017, and follow-up will end in December 2018.

**Conclusions:**

We expect the TD-CBT group to have better results compared to TAU on all posttreatment measures and that this improvement will be maintained during follow-up. This project could serve as a model for use in other areas or services of the National Health System in Spain and even in other countries.

**ClinicalTrial:**

International Standard Randomized Controlled Trial Number (ISRCTN): 58437086; http://www.isrctn.com/ISRCTN58437086 (Archived by WebCite at http://www.webcitation.org/6mbYjQSn3)

## Introduction

### Background and Rationale

Emotional disorders (EDs), including mood, anxiety, and somatization disorders, are a leading cause of disability and demand for primary care (PC) services [1,2]. According to Haro et al [1], 1-year prevalence rates in Spain for anxiety, depression, and somatization disorders are 6.2%, 4.4%, and 14.7%, respectively. In Spanish PC centers, the 1-year prevalence rates for depression, mood disorders, and mental disorders are 9.6%, 13.4%, and 31.2%, respectively [3]. These data indicate that approximately 1 out of 3 patients in the PC setting suffers from some type of mental disorder. In addition, comorbidity in these patients is high [4-6] and closely associated with poor quality of life [7], substance misuse, disability, and high health and social costs that rise in parallel with the increase in the number of comorbid disorders [8].

In 2001, the World Health Organization (WHO) estimated that the prevalence of mental disorders would continue to increase through the year 2020, thus imposing a significant social and economic burden on many countries around the world, especially in developed countries. For this reason, the WHO stressed the need to increase the number of specialized human resources to treat patients with EDs [9]. The most recent guidelines for depression and anxiety disorders published by the National Institute for Health and Clinical Excellence (United Kingdom) [10] recommend an evidence-based approach to identify the least intrusive but most effective interventions for the management of these disorders.

Cognitive behavioral therapy (CBT) is a highly efficacious and cost-effective approach to managing EDs; for this reason, it is currently considered the optimal therapy to treat these disorders. Although CBT is less expensive than most medical treatments, the costs can be further reduced by using a transdiagnostic group approach in which patients with different but related EDs (ie, they share certain commonalities, particularly high levels of anxiety and maladjusted thoughts) are grouped together [11]. This approach, known as transdiagnostic cognitive behavioral therapy (TD-CBT), addresses dysfunctional behaviors and thoughts with the aim of changing behavioral and thinking patterns. TD-CBT has been shown to be more effective than treatment as usual (TAU) in PC settings for the treatment of depression [12] and anxiety [13]; it has also been shown to be comparable or superior to many evidence-based psychological interventions for pain [14], especially when patients are referred by their general practitioners (GPs) [15].

Many patients with EDs are users of both PC and specialized care services. However, in many cases patients are misdiagnosed, with misdiagnosis rates of up to 78% for depression [16], 86% for panic disorder, 71% for generalized anxiety disorder (GAD), and 98% for social anxiety disorder [16,17]. Consequently, many patients may not receive appropriate treatment. Moreover, in the case of incorrect diagnoses, patients may also be subjected to costly, unnecessary, and potentially addictive and/or harmful (due to side effects) psychopharmacological treatments [16].

In Spain, the diagnosis of an ED is usually first made by the GP who must decide, in a very brief consultation (ie, less than 7 minutes), which psychoactive drugs to prescribe (if any) and whether or not the patient needs specialized care [18]. As a result of these time constraints, the most common treatments are pharmacological interventions. According to Codony et al [19], 39% of patients with anxiety disorders do not receive any treatment, one-third of patients receive medication alone, only 1% receive psychological therapy, and 27% receive combined psychological-medical therapy. These findings indicate that psychological treatment is underprescribed—often only as a last resort—in patients with an ED who seek help from their GP.

Despite the generalized underutilization of psychological treatments in the PC setting, in recent years, several countries—notably, the United Kingdom—have incorporated psychological services (including CBT) into the PC setting. In the last decade, the UK government developed and implemented a large-scale program entitled Improving Access to Psychological Therapies (IAPT) [20], designed to improve treatment of EDs among the general population. The results of that program have shown that CBT is as effective in routine PC as it is shown to be in research trials; importantly, these excellent results were achieved without any side effects, fewer relapses, and lower long-term economic and social costs than TAU [21]. The benefits of these psychological interventions include their effectiveness in reducing symptoms associated with depression and anxiety—effect size in pre-post treatment of 1.39 for anxiety problems and 1.41 for depression—and high recovery rates for those who completed treatment—74% for anxiety and 76% for depression. These benefits have helped to decrease the risk of relapse while maintaining long-term positive outcomes [22].

This efficient and novel way of providing patients with access to psychological therapies in the United Kingdom, where the most cost-effective treatment has improved the detection, diagnosis, and referral rates for these common mental health disorders, has generated intense interest in many countries as a treatment model, including in Spain. However, efforts are needed to implement this model in Spain.

### Objectives

#### Primary Objectives

Given the strong evidence base in favor of CBT versus TAU, together with the need to improve ED treatment in the PC setting in Spain, the major aim of the *Psicología en Atención Primaria* (PsicAP) study is to verify if a group-delivered TD-CBT for EDs is more effective and efficient than TAU in Spanish PC centers. If it is so, we want to compare the short- and long-term efficacy of TD-CBT versus TAU in the treatment of these disorders.  

#### Secondary Objectives

We also aim to compare the effect of these treatments on disability, quality of life, cognitive-emotional factors, and treatment satisfaction.

### Hypothesis

Regarding the primary objectives of this study, the experimental group as compared to the control group is expected to report a greater decrease (including pre-post treatment differences and decrease at 3-, 6-, and 12-month follow-up) in dysfunctional emotional symptoms and percentage of cases with probable EDs. The total scores of anxiety, panic, depressive, and somatic symptoms will be measured by the Patient Health Questionnaire (PHQ) [23,24]. In addition, regarding the secondary objectives of this study, we hypothesize that the results will prove that TD-CBT reduces disability and that it, consequently, improves quality of life. We expect that participants allocated to the experimental group will report decreased impairment on work, family, and social domains and an increase in physical, psychological, social, and environmental quality of life at posttreatment and at 3-, 6-, and 12-month follow-up. We also expect that, relative to the control group, participants in the experimental group will have higher levels of emotional regulation and lower scores on cognitive-emotional factors such as ruminative thinking, worries, metacognitions, and cognitive biases associated with EDs. Moreover, we expect the experimental group to report higher treatment satisfaction (see detailed explanation in the Outcomes sections below).

### Study Design

This is a two-arm—TAU control group and TD-CBT experimental group—single-blind randomized controlled trial (RCT); the psychologists responsible for assessing patients at the pre- and posttreatment evaluations are blinded to the treatment group. The experimental group will also include patients currently receiving TAU, which will be withdrawn if they are randomized to the TD-CBT group (see detailed explanation in the Interventions section below).

Treatment will be assessed at five time points: pretreatment (before randomization), posttreatment (at the end of the TD-CBT group session, 3-4 months after treatment initiation), and at three follow-up evaluations—3, 6, and 12 months after treatment finalization.

## Methods

### Study Setting

In Spain, as in many countries, PC is the first level of access to the public national health system. Following a communitarian vision of health care, PC is a level of care at which each patient's condition (and the course of disease) is monitored within the social environment. All PC centers are organized into basic structures of health—a health service concept established with the delimitation of territorial areas of health—in coordinated multidisciplinary teams (ie, GPs, nurses, pediatricians, social workers, and physiotherapists), with healing activities integrated with health promotion, disease prevention, etc. Every patient is assigned to a PC center of his or her basic structure of health and, therefore, to a team of carers at each PC center, a specialized center, and a hospital, covering all levels of possible need for health assistance. Nevertheless, clinical psychologists are not part of the PC team, but rather are located in specialized care units and hospitals.

The clinical trial is being conducted at the following 26 PC centers in Spain: Madrid (11 centers), Valencia (five centers), Biscay (one center), Albacete (one center), Mallorca (one center), Andalusia (two centers), Cantabria (one center), Navarra (two centers), and Galicia (two centers). These centers share many common characteristics, but several differences may exist between them, such as language (eg, Spanish [Castilian], Basque, Galician or Catalan) and other sociodemographic differences. As a result, this wide variety of locations makes these PC centers truly representative of Spanish society as a whole. The study may be extended to other centers in other cities around Spain given the interest in offering an alternative to TAU by PC health professionals.

### Target Population

The total sample is expected to consist of 1126 adults, all of whom will have a diagnosis of anxiety, depression, and/or somatic symptom disorders. Any anxiety disorder (eg, panic with or without agoraphobia, GAD, obsessive compulsive disorder, posttraumatic stress disorder, specific phobias, and social phobia) and other conditions included under somatization (eg, medically unexplained symptoms, chronic fatigue, or pain) may be included in the trial if they are not severe. We will test severity according to specific measures of disability and severity of the emotional distress group (see detailed explanation in the Outcomes section below).

Patients with a confirmed or suspected diagnosis of any of these disorders are invited by their GP to participate in the study. In addition, all patients who visit one of the participating PC centers will have the opportunity to be screened to see if they qualify for participation. If they meet the inclusion criteria, they are asked to participate and are included in the trial if they agree.

Before inclusion, patients are informed that they will be randomly assigned to either the control (TAU) or the experimental group (TD-CBT). Written informed consent is obtained from all patients. Participation is voluntary and confidentiality is guaranteed. The study protocol (ISRCTN58437086) has already been approved by the ethics and clinical research committee of the participating PC centers and by the Corporate Clinical Research Ethics Committee of Primary Care of Valencia (CEIC-APCV), the national research ethics committee coordinator. Participation in this trial does not involve any added risks to patients apart from the inherent risks associated with pharmacological treatment (TAU group only). The aim of this study is to maximize benefits and reduce potential harms (principle of proportionality) through TD-CBT.

### Inclusion and Exclusion Criteria

#### Inclusion Criteria

Any adult patient between 18 and 65 years of age seeking treatment for anxiety, depression, and/or somatic symptom disorder at any of the participating Spanish PC centers may be included in the study. Participation in the study is completely voluntary. For diagnosis, patients must meet predetermined cutoff points on the relevant subscale(s) of the PHQ [23,24]. The 15-item Patient Health Questionnaire (PHQ-15) (<10 points), the 9-item Patient Health Questionnaire (PHQ-9) (<12 points or original algorithm), the 7-item Generalized Anxiety Disorder (GAD-7) scale (<10 points), and the Patient Health Questionnaire-Panic Disorder (PHQ-PD) (modified algorithm) [25] are used to detect somatization disorders, major depressive disorder (MDD), GAD, and panic disorder, respectively (see Outcome sections below for details).

#### Exclusion Criteria

Patients over 65 years of age are excluded from the trial to avoid distorting the outcomes due to age-related difficulties; however, once the treatment has been validated, this important part of the population will be eligible for participation in these treatment program. Other exclusion criteria include the following: severe mood disorders (eg, bipolar disorder [GP diagnosis] or severe MDD: PHQ-9<23); substance abuse or dependence; any other severe mental disorder (eg, personality disorder); a history of frequent or recent suicide attempt(s); a high level of disability (Sheehan Disability Scale [SDS]<25) [26]; difficulty understanding the Spanish language; intellectual disability; difficulties in undertaking the group therapeutic process; or participation in another clinical trial.

### Interventions

#### Treatment-as-Usual Condition

The control group will receive TAU as provided by the GP at the Spanish PC center. This treatment has been described in previous research as nontreatment, standard treatment, pharmacological treatment, and/or practical advice by the GP delivered in routine care [27], focused on reducing negative emotional symptomatology.

The TAU is provided by the GP in their regular consultation, generally consisting of a face-to-face session (5-7 minutes) to assess the physical and/or psychological complaints of the patient. Also in this time, the GP will provide advice, medication—antidepressant, anxiolytics, or hypnotics—and/or onward referral to specialized care services. Importantly, since this is TAU, conditions are the same as in routine daily practice at the treatment center, without any modifications. If the GP recommends psychological treatment as part of the routine TAU, such patients are excluded from the final trial recruitment to avoid bias.

#### Experimental Condition

##### Rationale and Goals

The experimental group will receive TD-CBT group therapy. A well-documented and evidence-based therapeutic approach [11,22] that has been specially designed by Cano-Vindel [28] for the treatment of EDs in PC is used. This TD-CBT is focused on reducing negative emotional symptomatology in the short term using cognitive restructuring and behavioral management, which allows patients to continue without the use of medications in the long term. Any patient receiving TAU prior to study enrollment and then allocated to the TD-CBT group will be withdrawn from TAU. This means that patients in both groups may receive TAU before enrollment in the trial. Once patients are assigned randomly to the TD-CBT group, the GP is not permitted to provide or increase the TAU (ie, increasing pharmacotherapy), but may reduce or eliminate the medication if improvement is noted. It is expected that the pharmacological treatment in patients who are allocated to the TD-CBT group will be withdrawn by the GP as a result of improvement (ie, reduction) of the negative emotional symptoms due to TD-CBT.

##### Procedure and Schedule

Each participant will receive seven 90-minute sessions of TD-CBT group therapy—8-10 patients in each group, during approximately 12 weeks (3 months). This 12-week treatment period may be increased if necessary for scheduling purposes (ie, due to holiday periods) up to a maximum of 16 weeks (4 months). Sessions are facilitated by one clinical psychologist in a spacious and comfortable room at the PC center. The therapy is delivered with patients and therapist sitting in a circle. Space will also be needed for a relaxation session, and will include a CD player and mats. Paper material is provided for each session and patients may be required to bring a personal notebook. Table 1 shows the intervention schedule and the material provided in each treatment session.

The components of TD-CBT include the following: (1) Psychoeducation and information, designed to counteract misconceptions about emotions or EDs by providing correct information about EDs and treatment aims; (2) Relaxation, consisting of a series of techniques including training participants in progressive muscle relaxation, abdominal breathing, and visualization to reduce EDs and physical arousal; (3) Cognitive restructuring techniques to modify misconceptions about EDs; (4) Behavioral therapy to help participants learn to identify unadjusted emotions and behaviors in order to replace these with healthier ones; and (5) Relapse prevention to overcome difficulties and consolidate learning. All the components are shown in Table 2 with scheduling shown in Table 3.

**Table 1:** Schedule for the 7 treatment sessions.

Session	Schedule	Material provided
Session 1	First week	Presentation and group therapy rules Breathing and relaxation information sheet Breathing and relaxation self-register
Session 2	Second week	CD relaxation Cognitive restructure information sheet Activities self-register
Session 3	Third week	Down arrow exercise Cognitive biases information sheet
Session 4	Fifth week	Thought purification exercise
Session 5	Seventh week	Interpersonal solution problems sheet
Session 6	Ninth week	Reinforcement of previous activities
Session 7	Twelfth week	Relapse prevention exercise

**Table 2:** Components of the group TD-CBT^a^ protocol.

Psychological techniques	Components of each module	Session
Psychoeducation	Information about the following: • anxiety and mood state • emotional disorders • the group therapy • treatment components and the treatment aims • cognitive biases • the relationship between thoughts and emotions • Counteraction of the following: • misconceptions of emotions • misconceptions of emotional disorders	1 and 2
Relaxation	Abdominal breathing Training progressive muscle relaxation Visualization	1 and 2
Cognitive restructuring techniques	ABC Ellis Model Information about irrational and rational thoughts Exercises for the following: • detection and refutation of irrational thoughts with rational thoughts • detection of cognitive biases • to restructure cognitive biases • to provide positive self-instructions	3, 4, 5, 6, and 7
Behavior therapy	Behavioral activation Exposure therapy Social skills and assertiveness Solutions problems	5, 6, and 7
Relapse prevention	Acceptance of relapse Restructure of relapse	7

^a^TD-CBT: transdiagnostic cognitive behavioral therapy.

**Table 3:** Schedule of the sessions.

Psychological techniques	Session						
	1	2	3	4	5	6	7
Psychoeducation	X	X					
Relaxation	X	X					
Cognitive restructuring			X	X	X	X	X
Behavior therapy				X	X	X	X
Relapse prevention							X

##### Therapist Training

All therapists are experienced clinical psychologists. To work as a clinical psychologist in the National Health System in Spain, it is necessary to be certified as a Psychologist Specialist in Clinical Psychology. To obtain this certification, the therapist must have a university degree in psychology (4-5 years) and then must undergo a residency program—Internal Resident Program (IRP)—which is a postgraduate paid training system. The IRP consists of 4 years of work and training under the supervision of a specialist in the Spanish National Health System. In addition to this training, the clinical psychologists in this trial will also undergo a standardized training course conducted by a supervisor and trainer, who would have a PhD in clinical psychology. This training consists of studying the Therapist Manual, four Internet-based lessons on the content of each session, and one face-to-face session with the trainer. This must be completed before the clinical psychologist can provide any group therapy as part of the trial. All groups are supervised by one coordinator in each province. Follow-up sessions are conducted as necessary (ie, by request) to resolve any doubts after finalization of the training course.

To reduce attrition rates after the final posttreatment assessment, a clinical psychologist will telephone patients every 6 weeks posttreatment. During the 10-15-minute telephone consultation with patients in the TD-CBT group, the clinical psychologist will reinforce the psychological techniques taught during the group sessions and will follow up on the participants' emotional state. In the control group (TAU), the clinical psychologist will assess patients' emotional state and, if appropriate, recommend that they visit their GP.

### Primary Outcomes

#### The Patient Health Questionnaire

The PHQ [23] is a screening test derived from the Primary Care Evaluation of Mental Disorders (PRIME-MD) test, a self-reported measure of mental disorders designed for use in PC centers. We will use the Spanish version validated by Diez-Quevedo et al [24] to screen for EDs, using the sum scores of all the subscales independently, with some exceptions as explained below.

#### Somatization Disorder

The PHQ-15 was derived from the original PHQ studies and is commonly used to assess somatic symptom severity and the potential presence of somatization and somatoform disorders [29]. In the Spanish version, patients are asked to respond to 13 somatic symptoms, scored from 0 to 2 as follows: 0 (*not bothered*), 1 (*bothered a little*), or 2 (*bothered a lot*). Two items from the depression module (*sleep* and *tiredness*) will be added and scored as follows: 0 (*not at all*), 1 (*several days*), or 2 (*more than half the days* or *nearly every day*). The maximum score for the PHQ-15 will be 30. A probable somatization disorder is diagnosed when respondents score 2 points on at least five of the first 13 symptoms and the two items from the depression module, with a cutoff point of 10. Using this criterion, the PHQ-15 has a sensitivity of 78% and specificity of 71% for somatization disorder [30,31]. One study has shown that patients with somatization disorder (PHQ-15 diagnosis) utilize twice the amount of PC services as nonsomatizing patients, at twice the expense [30]. We used the Spanish language version of the original PHQ-15 included in the original PHQ [23].

#### Depression

The PHQ-9 [32] is a specific screening tool for depression in which participants use a 4-point Likert scale to respond to nine items (Fourth Edition of the Diagnostic and Statistical Manual of Mental Disorders [DSM-IV] criteria) about difficulties experienced during the prior 2 weeks. Using a cutoff of 10 points, the PHQ-9 has a sensitivity of 88% and specificity of 88% for depression. A score between 10 and 14 indicates minor depression, dysthymia, or moderate MDD; scores between 15 and 19 indicate moderately severe MDD; and scores between 20 and 27 indicate severe MDD. Participants who score between 20 and 23 will undergo a second-order assessment conducted by a clinical psychologist; in these cases, the Structured Clinical Interview for DSM Axis-I Disorders (SCID-I) scale for MDD (Spanish version) [33] is used to confirm the existence of severe MDD. Participants who score between 24 and 27 on the PHQ-9 and are confirmed by the SCID-I as having severe MDD are excluded from participation in the trial and referred again to their GP for referral to specialized care.

In a separate study [34], we studied the psychometric properties in a subsample of the larger PsicAP sample (n=178) and we found an optimal cutoff score of 12 on the PHQ-9, with a sensitivity and specificity of 84% and 78%, respectively. Nevertheless, using the original algorithm, the sensitivity and specificity values were 88% and 80%, respectively, thus recommending the use of the original algorithm due to its superior psychometric properties [34].

#### Panic Disorder

The PHQ-PD includes the DSM-IV-based panic disorder symptoms [23,24,35]. A diagnosis of probable panic disorder is made when the participant responds affirmatively to the first four items on the scale and to four or more of the symptoms. Nevertheless, when we studied the psychometric properties of this module in a subsample of the large PsicAP sample [25], we obtained better sensitivity (77%) and specificity (72%) using a modified algorithm as follows: when participants respond affirmatively to the first screening item, to one of the three items on the next scale, and to four or more items of the somatic symptoms [25].

#### Anxiety

The GAD-7 scale is used to measure GAD and other anxiety disorders [36]. In this scale, patients rate the frequency of anxiety symptoms during the past 2 weeks. Total scores of 5, 10, and 15 indicate mild, moderate, and severe anxiety, respectively. The maximum score is 21 and the cutoff score is 8—a score of at least 2 on the first question, plus three more items. Using a cutoff of 10, the GAD-7 scale has a sensitivity of 89% and a specificity of 82% for GAD [36].

In our study, we used the validated Spanish version of the GAD-7 scale [37] instead of the PHQ items related to anxiety disorders. Factor analysis of the Spanish version of the GAD-7 scale has shown that all items in the GAD-7 scale load onto one factor and the scale uses a cutoff score of 10 to detect GAD [37]. In addition, when we evaluated the psychometric properties of this GAD-7 version in our PC subsample, an optimal cutoff score of 10 was obtained, showing a sensitivity of 87% and a specificity of 78% [34].

#### Eating Disorders and Alcohol Abuse

The PHQ also contains screening items to detect eating disorders such as bulimia nervosa or binge eating disorder and to check for the presence of alcohol abuse. If items 6(a)-(c) and 8 are scored as a “yes,” the score is considered positive for bulimia nervosa; for binge eating disorder, the criteria is the same except for item 8 (either “no” or left blank). The Spanish version of the PHQ was used, which has a sensitivity of 92% and a specificity of 98% for any eating disorder [24]. Alcohol abuse is detected if the patient answers “yes” to any of items 10(a)-(d). The Spanish version of the PHQ [24] has a sensitivity of 76% and a specificity of 99% for probable alcohol abuse or dependence. Participants who have positive scores on these subscales are briefly interviewed by a clinical psychologist to confirm the diagnosis. If they present with an eating disorder, alcohol abuse or dependence, or probable personality disorder they are excluded from participation in the trial and referred to their GP for referral to specialized care.

### Secondary Outcomes

#### Disability

The Sheehan Disability Scale [26] is a 5-item self-report scale that measures subjective impairment during the past month in three key areas: work, family, and social functioning. Two additional questions on the SDS are designed to assess the level of stress and perceived social support in the past week. We used the Spanish version developed by Bobes et al [38], which has shown good reliability and validity. The first four items are rated on an 11-point Likert scale from 0 (*no dysfunction*) to 10 (*maximum dysfunction*). The fifth item uses the same scale but is expressed in percentages from 0% (*no social support*) to 100% (*ideal social support*). Scores of 1-3, 4-6, and 7-9 indicate mild, moderate, or high disability, respectively. Overall scores of 25 or more indicate a high level of disability. In these cases, a psychologist will ask participants the following three questions before excluding them from the study: (1) Are you on sick leave? (2) Can you do the housework? and (3) Can you engage socially?

#### Quality of Life

The World Health Organization Quality of Life Instrument-Short Form [39] is a 26-item questionnaire used to measure perceived quality of life in four domains: physical, psychological, social, and environmental. This instrument is used worldwide, including in Spain [40], and shows good psychometric properties, reliability, and validity [41].

#### Cognitive-Emotional Factors

Several subscales or short questionnaires are used to evaluate brooding, worries, and cognitive biases. The 5-item Brooding Scale of the Ruminative Response Scale [42] has been validated in Spanish with good reliability and validity [43]. The Spanish version of the Penn State Worry Questionnaire [44,45] is used, specifically an 8-item version similar to the abbreviated version (PSWQ-A)[46]. A brief 5-item version of the Inventory of Cognitive Activity in Anxiety Disorders (IACTA) will be used to assess attentional biases.

We will use the 10-item validated Spanish version of the Emotion Regulation Questionnaire, which has been shown to have good reliability and validity [47,48] to assess emotion regulation. We will use the 6-item metacognitive beliefs subscale of the Metacognitions Questionnaire [49] to assess metacognitions. This has been validated in Spanish with good reliability and validity [50].

Note that the Spanish version of the PSWQ-A has not been validated yet and the IACTA is under review. However, the reliability and validity of these scales and subscales were recently completed in a subsample of the large PsicAP sample and results are expected to be published soon.

#### Treatment Satisfaction

All participants are surveyed to assess their level of satisfaction with the treatment received; participants rate their satisfaction on a scale from 0 (*high dissatisfaction*) to 10 (*high satisfaction*).

### Sample Size

The minimum sample size required to obtain a significant result has been calculated with the SPSS version 21.0 Sample Power program (IBM Corp). The study will include at least 563 patients in each group for a total of 1126 patients, assuming a 20% attrition or dropout rate. With this sample size, the result will be statistically significant (85% statistical power) when comparing both groups, even if they differ by one point only on the subscales of the PHQ measures, with a standard deviation of 5. This will enable us to conclude that the result is different for each group with a 95% confidence level. As previous studies have reported [11], we expect that the rate of loss to follow-up will be considerable, despite the strong study design, which includes telephone follow-up to reduce attrition. Consequently, this is likely to be an important limitation of our study.

### Patient Recruitment

Patient recruitment is carried out in two phases, as follows.

#### First Phase

Patients who present with signs or symptoms of anxiety or depression, negative emotions, or physical symptoms for which there was no clear biological basis are preselected by the GP for possible participation. Patients currently being treated with antidepressants, anxiolytics, and/or hypnotics may also be invited to participate in the study. The GP will explain the clinical trial to these patients and ask if they wish to participate. Prior to study participation, patients will receive written and oral information in the patient information sheet about the content and extent of the planned study. This includes information about the potential benefits and risks for their health. All patients who agree to participate are required to sign the informed consent form.

#### Second Phase

All patients who consent to participate in the trial (ie, have signed informed consent forms) are contacted by a clinical psychologist, who will then schedule an appointment to complete the aforementioned screening questionnaires. Only patients who meet the study inclusion criteria on the PHQ subscales—PHQ-15, PHQ-9, PHQ-PD, and GAD-7—are enrolled, using the cutoff scores and algorithms described above. All other patients are referred back to their GP for alternative treatments.

### Randomization

Participants are randomly assigned after informed consent or assent is obtained by a blinded researcher using a computer-generated allocation sequence, assuring that the groups are comparable (ie, without differences in key baseline measures). Each group will include 8-10 patients randomly allocated either to the experimental group (TD-CBT) or to the control group (TAU). They receive this allocation information via email from a graduate student trainee affiliated with the project. The email also provides login and website information for the allocated intervention. One clinical psychologist is assigned to the TD-CBT group; the clinical psychologist involved in the pre- and posttreatment assessment phases will not participate in the TD-CBT therapy. Data managers and statisticians are blinded to the treatment allocation.

### Data Collection

After providing written informed consent, the participants are registered in the treating center. Pre- and posttreatment assessments are carried out using computerized self-reported screening tests. All pretreatment assessments are performed at the treating PC center after scheduling an appointment with the clinical psychologist. A computer with Internet access is used to collect data. All data are stored on a general virtual website (surveymonkey.com). At all posttreatment follow-up assessments, the same instruments will be completed in person at the treating center. However, if necessary, we will send the participant a link by email to enable the patient to complete the computerized measures at home. Patients are contacted by phone to encourage completion of the questionnaires. Moreover, those patients that discontinue or drop out of treatment will still be invited to complete the posttreatment follow-up assessments, particularly the first posttreatment assessment.

### Data Analysis

Analysis will be carried out using SPSS version 21.0 (IBM Corp). Intention-to-treat (ITT) analysis will be performed. The ITT analysis will include all randomized patients in the groups to which they were randomly assigned. Analysis will take into account noncompliance, protocol deviations, dropouts, and anything else that happens after randomization. Using the ITT approach will enable us to include situations likely to occur in actual clinical practice. This “real-life” analytical approach allows us to assess the prognostic balance resulting from the original random treatment allocation, thus providing a more accurate estimation of treatment effect. Missing-data analysis will be computed using Student's *t* test and chi-square tests. Variables included in the analysis will be severity level, gender, and age; this will allow us to ascertain whether unexpected missing data due to participant dropout are related to chance or not

The two randomized groups will be compared in the treatment period; posttreatment; and at 3, 6, and 12 months after treatment finalization. In addition, within-subject comparisons will be analyzed, contrasting pretreatment and posttreatment scores. The within-group and between-group differences will be examined using mixed-effect models, since these are considered more accurate than univariate and multivariate repeated measures of variance [51]. Group differences will be analyzed after controlling for baseline levels, gender, age, and treatment center. Additionally, we will estimate the percentage of patients in each group who experience a 50% decrease in the number of clinical symptoms and scores by one standard deviation, as well as the percentage of cases with a probable ED before and after receiving treatment (according to cutoff criteria).

The TD-CBT therapy will be considered effective if average scores on ED symptoms—anxiety, depression, and somatic symptoms—of patients who receive treatment are significantly lower than average scores of the control group and if effect sizes (Cohen  *d*) are low to medium. Both groups will be compared to test for differences in level of disability—work, family, and social functioning—quality of life, and treatment satisfaction.

### Ethics and Dissemination

#### Research Ethics Approval

This is a multicenter RCT with medication (N EUDRACT: 2013-001955-11; protocol code: ISRCTN58437086) promoted by the Psicofundación (The Spanish Foundation for the Promotion of the Scientific and Professional Development of Psychology). The trial was approved by the CEIC-APCV—the national research ethics committee coordinator—and the Spanish Medicines and Health Products Agency. Approval was received by both agencies in November 2013, prior to study initiation in December 2013.

The CEIC-APCV approved the trial in three centers in the autonomous communities of Valencia (one center), the Balearic Islands (one center), and Castilla-La Mancha (one center). The study was also approved by the local ethics committees of the first three centers: the CEIC-APCV, the Clinical Research Ethics Committee of the Hospital Universitario de Albacete (CEIC-HUA), and the Clinical Ethics Committee of the Balearic Islands (CEIC-IB).

#### Protocol Amendments

Six protocol amendments have been presented during the course of this trial.

##### Amendment 1

One PC center was added to the autonomous communities of the Basque Country and was approved by the Clinical Research Ethics Committee of Euskadi (CEIC-E). In addition, a substudy—substudy 1—was approved in order to conduct the study of the psychometric properties of the PHQ subscales of the PHQ-9, PHQ-PD, and GAD-7 with 15% of the larger sample. This substudy has been conducted in four PC centers located in the autonomous communities of Valencia (one center), the Balearic Islands (one center), the Basque Country (one center), and Castilla-La Mancha (one center). The substudy was also approved by the first four local ethics committees: the CEIC-APCV, the CEIC-HUA, the CEIC-E, and the CEIC-IB.

##### Amendment 2

Nine centers located in the Community of Madrid were added to the study. The Clinical Research Ethics Committee of Madrid approved this amendment, as did the national ethics committee, the CEIC-APCV.

##### Amendment 3

One PC center was added to the group of centers in the autonomous community of Valencia. This center thus became a full participant in the trial and substudy 1, bringing the number of PC centers in substudy 1 to five. In addition, several changes to the first version of the protocol were made, including the use of the SCID-I to confirm severe MDD and questions to confirm high disability on the SDS, as described above. Also, new researchers were added to the study. The amendment was approved by the national ethics committee—the CEIC-APCV—and by the relevant local ethics committees.

##### Amendment 4

Three PC centers, two in Andalusia and one in Cantabria, were added to the list of participating centers. In addition, substudy 2 was presented, which is a study of the cost-efficiency measures that are conducted in the PC centers in Madrid and Valencia. Several changes to the next version of the protocol were made, including the telephone follow-up posttreatment (see Therapist Training section above). Finally, new researchers were added to the study. The amendment was approved by local ethics committees—the Clinical Research Ethics Committee of Córdoba and the Clinical Ethics Committee of Cantabria—and the national ethics committee, the CEIC-APCV.

##### Amendment 5

Five PC centers were added to the autonomous communities of Madrid (two centers) and Valencia (three centers) to conduct the trial. Also, new researchers were added to the study. The amendment was approved by the local ethics committee—the Clinical Research Ethics Committee of Madrid—and the local and national ethics committee, the CEIC-APCV.

##### Amendment 6

Six PC centers were added to the autonomous communities of Catalonia (two centers), Galicia (two centers), and Navarra (two centers) to conduct the trial. Also, new researchers were added to the study. The national legislative norms have been modified in Spain and now only one national ethics committee is required for RCTs. As a result, this amendment was approved by the national ethics committee, the CEIC-APCV. One new substudy was also presented. Substudy 3 is a modification of the protocol design (ie, stepped-wedge trial design), which will be conducted in two PC centers in Barcelona (Catalonia). In addition, a change to the next version of the protocol was made with regard to using the 4-item Patient Health Questionnaire (PHQ-4) to detect EDs in PC centers by the GP; the aim is to reduce misdiagnoses of EDs and to accelerate referral to the clinical psychologist in the second phase of the recruitment process. This will allow us to determine if the ultrashort measure of the PHQ-4 is an appropriate tool to help GPs to detect EDs and to reduce the large number of false negatives. If results are as expected, this may lead to a proposal for a new referral model in Spanish PC centers.

#### Consent

Regarding patient informed consent, prior to study participation, all patients receive written and oral information in the patient information sheet about the content and extent of the planned study. This includes information about the potential benefits and risks to their health. Patients who agree to participate are required to sign the informed consent form. In the case of patients who withdraw from the study, all data will be destroyed or the patient will be asked if he/she agrees to allow the use of existing data for analysis in the study.

Patient participation in the study is completely voluntary and participants can withdraw at any time with no need to provide reasons and without negative consequences for their future medical care. The protocols used in this study pose no risk whatsoever to the participants. TD-CBT is noninvasive at the cognitive level, except with regard to learning or teaching.

#### Confidentiality

The study is conducted in accordance with Spanish data security law. All professionals participating in the study agreed to adhere to the Helsinki Declaration and to Spanish law. All health care professionals participating in the study are required to sign a form indicating their agreement to adhere to the above-mentioned declaration and Spanish law.

The patient names and all other confidential information fall under medical confidentiality rules and are treated according to Spanish data security law. The patient questionnaires are collected by the researchers (not nurses) and mailed by secure transport to the study center in Madrid. All study-related data and documents are stored on a protected central server and saved in an encrypted database.

The project complies with current guidelines in Spain and the European Union for patient protection in clinical trials with regard to the collection, storage, and keeping of personal data. Only direct members of the internal study team can access the data.

#### Access to Data

The study data are only available upon request. The name(s) of the contact person(s) to request data are available upon request to all interested researchers. Legal and ethical restrictions make data available upon request and are in accordance with the nature of the data collection.

The CEIC-APCV have some availability restrictions as part of the legal and ethical control of data from an RCT with medication.

Data are available from the promoter (Spain) for researchers who meet the criteria for access to confidential data. Interested researchers should contact Psicofundación (The Spanish Foundation for the Promotion of the Scientific and Professional Development of Psychology) at the registered office at Calle Conde de Peñalver, 45, 5o izquierda, 28006 Madrid.

#### Concomitant Care

No concomitant care has been registered.

#### Dissemination Plans

One of the major objectives of this trial is to convince public health care administrators to implement, once the efficacy has been proven, these evidence-based psychological treatments for EDs in the PC, under the guidance of clinical psychologists. Given the current situation of the National Health System in Spain, the number of clinical psychologists will need to be increased and positions will need to be created at Spanish PC centers for clinical psychologists.

## Results

This study was initiated in December 2013 and will remain open to new participants until recruitment and follow-up has been completed. We expect all posttreatment evaluations to be completed by December 2017, and follow-up will end in December 2018.

## Discussion

### Principal Findings

Emotional disorders are common in the community, highly comorbid, and they often affect personal functioning and well-being. According to the WHO [9], mental disorders will generate a large social and economic burden in all countries in the year 2020. This organization estimates that the current number of specialized human resources to treat these disorders is insufficient [9]. Supported by international guidelines such as the UK National Institute for Health and Clinical Excellence [10], the WHO recommends implementation of evidence-based interventions to treat EDs. Psychological treatments such as CBT are considered the treatment of choice for EDs given their relative cost-effectiveness in the long term versus pharmacological treatment [52-54]. Moreover, published reports indicate that CBT may be even more effective for EDs when a transdiagnostic approach is used (TD-CBT) [11,55].

In addition to the WHO report described above, other studies have also found that human resources available for treatment of EDs, especially clinical psychologists in European countries, are inadequate [56]. Several reports have demonstrated that insufficient human resources in Spain can lead to misdiagnosis and malpractice [18,19]; moreover, this deficit of trained staff places increased demands on an already oversaturated health care system. Despite this lack of resources, several Spanish studies have found that CBT group therapy is highly efficacious in treating depression in both the short and long term [57], as well as for other EDs [58].

Given the limited availability of clinical psychologists in specialized settings, their scant presence in PC centers—the gateway of patients to the health care system—is not surprising. According to Serrano-Blanco et al [3], approximately 1 out of 3 patients in the PC setting has some type of ED. This high prevalence, together with the shortage of clinical psychologists, underscores the need to increase the availability of these specialists in PC. This is especially true given the substantial evidence supporting the effectiveness of CBT.

At present, our group is carrying out this novel project in Spain to validate the cost-efficiency implementation of psychological treatment in PC centers. Currently, patients in PC centers are primarily treated by GPs and psychiatrists. However, we advocate the use of a collaborative, stepped-care, PC-based psychological intervention to reduce anxiety, depression, somatizations, and disability while simultaneously increasing quality of life. The proposed psychological intervention—TD-CBT—is a promising intervention delivered by clinical psychologists following a rigorous scientific protocol designed to provide optimal care of patients with EDs.

If the results of this clinical trial are positive, as we expect, these outcomes will provide further support in favor of incorporating clinical psychologists into the PC setting to administer TD-CBT group therapy for EDs as the treatment of choice. Implementation of this model will likely improve treatment adherence and, consequently, lower the health care burden of treating EDs. In addition, again assuming that the results are as expected, this will provide further support to the growing body of evidence pointing to the value of TD-CBT group therapy in PC settings. We fully expect that this intervention will improve the health of patients in the experimental group and will increase the quality of life and well-being of both patients and relatives. The ability to offer TD-CBT group therapy would also help to form therapy groups consisting of patients with several comorbid disorders, thus further helping in quick group formation. It will be interesting to study what the implications could be of the cost-efficiency of this treatment modality.

One of the major interests of this project is the need to increase the number of clinical psychologists in the Spanish public health care system. The Spanish government recently acknowledged that the public health system needs an additional 7200 clinical psychologists to reach the European average of 18 per 100,000 people. If the results of this study are similar to those achieved in studies conducted in other countries (eg, Australia [59,60], Great Britain [22], Norway [61], and the United States [62]), then this would provide further empirical evidence in support of more interventions of this type and thus more clinical psychologists. Furthermore, the experience gained in this study will enable us to easily train other clinical psychologists by applying the manualized TD-CBT program used in this trial, with no need to further train other professionals.

### Study Limitations

This study has several limitations related to the functioning of Spanish PC centers. GPs are the first point of contact for patients with EDs and these physicians have only approximately 5-7 minutes to evaluate, diagnose, and offer these patients TAU or to recommend participation in this clinical trial. Logically, the recruitment process for this trial could be negatively affected by these time constraints, especially considering that the GP must make a concerted effort to motivate and recruit a large number of patients in a relatively short period of time. Another limitation is that the PC centers have not been randomly selected. Despite this limitation, it is important to highlight two of the main strengths of this study: the large sample of patients (>1000 patients) and the large number of PC centers (>20) distributed in a wide geographic range all over Spain.

Another study limitation is that the TD-CBT intervention is scheduled to last from 12 to 14 weeks and can be affected by several factors (eg, vacations and availability of participants) beyond our control. Thus, the follow-up assessments—3, 6, and 12 months posttreatment—might be performed at different time points. However, we are making every effort to ensure that the duration and timing of TD-CBT treatment and follow-up assessments are homogenous among the groups and coincide with the assessments of the TAU intervention, but we cannot rule out the possibility of variability.

Additionally, as a limitation similar to other studies of this nature, we expect a considerable rate of loss to follow-up. However, the study design includes measures—primarily telephone and email follow-up—aimed at reducing the rate of loss. Nevertheless, it seems likely that many patients in both groups will not complete all follow-up assessments. It is likely that some patients will discontinue or drop out of treatment. However, we are registering the number of sessions that patients in the TD-CBT group attend in order to determine the mean number of sessions attended; this may improve our outcome measures, particularly if it shows that some sessions are more effective than others. Nevertheless, in the TAU group, we are not registering the number of sessions, as this is an inherent condition of TAU. As said above, regarding those patients that drop out of either group, we still invite them to complete the posttreatment follow-up assessments.

An important aim of this project is to improve the current referral model, in which GPs refer patients with suspected EDs to specialist services. This new model seeks to increase interaction between GPs, clinical psychologists, and specialized centers through a new referral system based on the implementation of a stepped-care model. The use of validated instruments to achieve more accurate diagnoses, together with the use of more effective treatments, will potentially decrease the number of GP visits, thereby helping to optimize current PC resources. In this regard, the role of the clinical psychologists participating in this clinical trial is crucial.

Once recruitment is completed, patients will continue to receive care from their primary care GP, who is easily accessible, without cost to the patient. We are confident of the success of this treatment program and, if successful, it will add another tool—TD-CBT—to the available resources for treating EDs. Clinical guidelines may need to be updated to reflect the study's outcomes. We expect this study to yield valuable data about the short- and long-term efficacy of TD-CBT group therapy for EDs applied by clinical psychologists in a PC setting. Our findings could help design stronger and more effective public health strategies and treatments, leading to better care of patients with these disorders.

Finally, it is important to note that this project and its design are novel in the PC setting in Spain. If the results are as expected, this project could serve as a model for use in other areas or services of the National Health System in Spain and even in other countries.

## Abbreviations

CBT: cognitive behavioral therapy
CEIC-APCV: Corporate Clinical Research Ethics Committee of Primary Care of Valencia
CEIC-E: Clinical Research Ethics Committee of Euskadi
CEIC-HUA: Clinical Research Ethics Committee of the Hospital Universitario de Albacete
CEIC-IB: Clinical Ethics Committee of the Balearic Islands
DSM-IV: Fourth Edition of the Diagnostic and Statistical Manual of Mental Disorders
ED: emotional disorder
GAD: generalized anxiety disorder
GAD-7: 7-item Generalized Anxiety Disorder
GP: general practitioner
IACTA: Inventory of Cognitive Activity in Anxiety Disorders
IAPT: Improving Access to Psychological Therapies
IRP: Internal Resident Program
ITT: intention to treat
MDD: major depressive disorder
PC: primary care
PHQ: Patient Health Questionnaire
PHQ-4: 4-item Patient Health Questionnaire
PHQ-9: 9-item Patient Health Questionnaire
PHQ-15: 15-item Patient Health Questionnaire
PHQ-PD: Patient Health Questionnaire-Panic Disorder
PRIME-MD: Primary Care Evaluation of Mental Disorders
PsicAP: Psicología en Atención Primaria
PSWQ-A: abbreviated version of the Penn State Worry Questionnaire
RCT: randomized controlled trial
SCID-I: Structured Clinical Interview for DSM Axis-I Disorders
SDS: Sheehan Disability Scale
TAU: treatment as usual
TD-CBT: transdiagnostic cognitive behavioral therapy
WHO: World Health Organization
